# Who Benefited Most from the Internet-Based Conversational Engagement RCT (I-CONECT)? Application of the Personalized Medicine Approach to a Behavioral Intervention Study

**DOI:** 10.14283/jpad.2024.41

**Published:** 2024-02-15

**Authors:** Chao-Yi Wu, K. Yu, S. E. Arnold, S. Das, H. H. Dodge

**Affiliations:** 1 Neurology, Massachusetts General Hospital (MGH), Harvard Medical School, Boston, USA; 2 Neurology, Oregon Health & Science University (OHSU), Portland, USA; 3 149 13th floor, 10-003C, Boston, MA USA

**Keywords:** Responders, treatment heterogeneity, delay in cognitive decline, random forest, SHapley Additive exPlanations (SHAP)

## Abstract

**Background:**

Many Alzheimer’s Disease (AD) clinical trials have failed to demonstrate treatment efficacy on cognition. It is conceivable that a complex disease like AD may not have the same treatment effect due to many heterogeneities of disease processes and individual traits.

**Objectives:**

We employed an individual-level treatment response (ITR) approach to determine the characteristics of treatment responders and estimated time saved in cognitive decline using the Internet-based Conversational Engagement Clinical Trial (I-CONECT) behavioral intervention study as a model.

**Design and Setting:**

I-CONECT is a multi-site, single-blind, randomized controlled trial aimed to improve cognitive functions through frequent conversational interactions via internet/webcam. The experimental group engaged in video chats with study staff 4 times/week for 6 months; the control group received weekly 10-minute check-in phone calls.

**Participants:**

Out of 186 randomized participants, current study used 139 participants with complete information on both baseline and 6-month follow-up (73 with mild cognitive impairment (MCI), 66 with normal cognition; 64 in the experimental group, and 75 in the control group).

**Measurements:**

ITR scores were generated for the Montreal Cognitive Assessment (MoCA) (global cognition, primary outcome) and Category Fluency Animals (CFA) (semantic fluency, secondary outcome) that showed significant efficacy in the trial. ITR scores were generated through 300 iterations of 3-fold cross-validated random forest models. The average treatment difference (ATD) curve and the area between the curves (ABC) were estimated to measure the heterogeneity of treatment responses. Responder traits were identified using SHapley Additive exPlanations (SHAP) and decision tree models. The time saved in cognitive decline was explored to gauge clinical meaningfulness.

**Results:**

ABC statistics showed substantial heterogeneity in treatment response with MoCA but modest heterogeneity in treatment response with CFA. Age, cognitive status, time spent with family and friends, education, and personality were important characteristics that influenced treatment responses. Intervention group participants in the upper 30% of ITR scores demonstrated potential delays of 3 months in semantic fluency (CFA) and 6 months in global cognition (MoCA), assuming a 5-fold faster natural cognitive decline compared to the control group during the post-treatment period.

**Conclusions:**

ITR-based analyses are valuable in profiling treatment responders for features that can inform future trial design and clinical practice. Reliably measuring time saved in cognitive decline is an area of ongoing research to gain insight into the clinical meaningfulness of treatment.

## Introduction

**A** major challenge for Alzheimer’s disease (AD) clinical trials is the large individual heterogeneity in disease, its progression, and its responses to treatment. Many pharmaceutical and behavioral clinical trials have failed to demonstrate treatment efficacy, possibly due to insensitivity of analyses to such heterogeneities. Recent successful trials demonstrated trial efficacy by comparing group means of cognition and functional outcomes (1, 2), yet not all the participants might benefit to the same magnitude (3, 4). Identifying the characteristics of treatment responders in a clinical trial is critical because it can assist in targeting the treatment population, estimate the required sample size in subsequent studies (5), increase the chances of observing meaningful effects, and inform clinical implementation. For early-phase AD trials, the profile of treatment responders also becomes critical for understanding mechanisms of change and driving the design of the next trial phases (6, 7). With well-defined responder profiles, the right treatment can be prescribed for the right patient and treatments can be prescribed to maximize benefit and minimize adverse events, thereby facilitating precision medicine while maintaining safety.

“Responder”, in the context of a randomized controlled trial (RCT), has been discussed in the regulatory guidance from FDA (8):

“…*.it is appropriate for a critical distinction to be made between the mean effect seen (and what effect might be considered important) and a change in an individual that would be considered important, perhaps leading to a definition of a responder…. There may be situations where it is more reasonable to characterize the meaningfulness of an individual’s response to treatment than a group’s response* (8).”

This guidance highlights two important messages. First, a person’s response to the treatment should be characterized for that individual rather than comparing the placebo and experimental group responses. Second, the magnitude of treatment effect should be important and meaningful for an individual. However, traditional responder analysis falls short in addressing these critical messages. Conventional methods rely on measuring the change in outcome within the treatment group to classify responders versus non-responders. This approach implies that any improvement in the outcome is solely attributed to the treatment (9). Yet, there are many situations where improved scores could occur, such as learning effects, measurement errors, other health changes, or even seasonal influences (9). Even when a placebo group is present, the conventional responder analysis typically adjusts an individual’s treatment effect based on the placebo effect as a group, rather than the individual placebo effect. This limitation stems from the design of RCT, where an individual cannot simultaneously be assigned to both treatment and placebo arms. Even if a participant demonstrates improvement due to the treatment, it remains uncertain whether the same participant would experience similar or even more enhancement with the placebo (10), a concept known as negative responders. Conversely, if a participant does not exhibit a positive response to the treatment, there is uncertainty about whether the same participant might experience a decline when subjected to the placebo. By not comparing an individual’s improvement or decline under both experimental and placebo conditions, the conventional approach runs the risk of misclassifying responders and miscalculating the magnitude and thus, meaningfulness of an individual’s treatment responses.

A more suitable method for identifying responders would involve comparing a participant’s outcome when receiving both treatment and placebo. Many methodologies have been introduced to achieve this goal (3, 11). A two-step approach was proposed by Zhao and colleagues (3) to quantify heterogeneity of treatment responses and identify responders. It first builds prediction models for an outcome of interest separately for the experimental and placebo arms using baseline participant characteristics as predictors. Then, an individual-level treatment response (ITR) score is estimated for each participant, representing the difference between the predicted outcomes under the experimental and placebo arms. This approach gives information on how much a participant benefits from the treatment while controlling for the benefit he or she could receive from the placebo (and vice versa). Ultimately, this approach allows for the evaluation of treatment response heterogeneity and responder profiles.

In this study, we applied an ITR approach to the Internet-based conversational engagement clinical trial (I-CONECT, NCT02871921) data. The I-CONECT is a social interaction cognitive stimulation intervention that has shown efficacy in improving cognition in socially isolated older adults (12, 13). In this paper, we presented a series of analytics to identify subgroups of participants with differential potential for treatment responses. The analysis focused on two cognitive outcomes that exhibited significant intervention efficacy in our study as previously described (13): the primary outcome (Montreal Cognitive Assessment, MoCA (14), measuring global cognitive function) and secondary outcome (Category Fluency Animals, CFA (15), measuring semantic fluency). We further conducted a proof-of-concept analysis to see if I-CONECT could save time on cognitive decline in treatment responders (16). This sequence of analyses presents a rigorous method for constructing responder profiles and offers valuable insights to guide trial enrichment strategies and facilitate personalized medicine for future AD trials and clinical practice.

## Methods

### I-CONECT

The I-CONECT is a single-blind, multi-site (Portland, Oregon; Detroit, Michigan), randomized controlled trial. The experimental group received a 30-minute video chat with study staff 4 times per week for 6 months (high dose), and then 2 times per week for an additional 6 months (maintenance dose). Both experimental and control groups received weekly 10-minute check-in phone calls. Participants were socially isolated older adults aged ≥ 75 years old, with or without MCI. We applied the ITR approach to baseline and 6-month follow-up data (the primary analysis of the trial) for the purpose of showing the application procedures of the ITR approach in the current analysis. We previously presented the trial results separately for the MCI and normal cognition groups and showed that efficacy was shown in MoCA among the MCI and CFA among those with normal cognition. However, for simplicity, we combined both cognitive groups in the current analysis but included a cognitive status indicator as a covariate (discussed later) to secure a larger sample size. The detailed inclusion/exclusion criteria, the study protocol and topline results were published (13, 17). The I-CONECT study was approved by the institutional review board (IRB) at the Oregon Health & Science University (OHSU) (STUDY00015937) using a single IRB process.

### Primary and secondary outcomes

The primary outcome was the MoCA, a measure of global cognition. This was administered at baseline and 6 months follow-up. The study was ongoing at the time the COVID-19 pandemic in-person contact restrictions began (March, 2020). 56 participants received in-person MoCA assessment at both baseline and 6 months, 50 had in-person visits at baseline but virtual visits at 6 months, and 34 had virtual visits at both baseline and 6 months. In lieu of the in-person MoCA, the Telephone MoCA (T-MoCA) was administered at virtual assessments. The full MoCA score was imputed using the T-MoCA score at baseline and 6-month follow-up. The COVID-19 related study modification and MoCA imputation approaches were published elsewhere (17).

CFA test was administered at baseline and 6-month follow-up either in-person (before the COVID-19) or via phone call (during the COVID-19). Administration of this test is similar for telephone vs. in-person assessments and therefore, we combined the telephone and in-person test scores. Semantic memory measured by Craft Story Immediate and Delayed Recall Tests were also secondary outcomes, but the test showed efficacy only at 12-month follow up among the MCI subjects. Therefore, it is not examined in the current ITR analysis.

### Social and clinical characteristics

The following variables, which are assumed to have the potential to influence responses to the intervention, are included: age, sex, education, race (White, Black or other non-White), living arrangement (alone or with others), depressive symptoms (Geriatric Depression Scale-15, GDS- 15) (18), and personality (the NEO Five-Factor Inventory including agreeableness, conscientiousness, extraversion, neuroticism, and openness domains (19)). These variables were assessed at baseline and treated as predictors in the models. We also included a self-reported amount of social interactions and time out-of-home per week (proximate indicators of socialization) in the models. All participants received weekly check-in phone calls and were asked to report the time spent in-person, video-chat, and text-email with family, friends, and others on a Likert-scale (converted to an hour scale: 0–7 to 0, 0.25, 0.75, 1.5, 2.5, 3.5, 4.5, 5 hours) (20). Time out-of-home per week was reported on a Likert-scale and was converted to an hour scale (1–8 to 0, 0.25, 0.75, 1.5, 2.5, 3.5, 4.5, 5 hours). Self-perceived health status (Likert-like score 1–5, higher is worse) was also included. The first 4 weeks of weekly phone check-in responses on the above social and health variables were averaged for the analysis.

### Statistical Analysis

#### Individual-level treatment response (ITR) score

For each cognitive outcome, we conducted the following procedure 900 times (300 rounds of 3-fold cross-validated random forest models). A random forest model was fitted onto experimental and control groups separately to model 6-month cognitive test scores using training sets (139*67% ≈ 93 participants for each fold in each iteration). Next, the two models were used to predict 6-month cognitive test scores for all samples in the testing/ holdout set (139*33% ≈ 46 participants for each fold in each iteration). The procedure can be described as follows. Let *Y*_*(k)*_^*i*^ be the cognitive outcome for participant i and *k*∈*{1:Treatment,0:Placebo}.* Let *μ*_*k*_ *(Z*^*i*^*)=E(Y*_*(k)*_^*i*^*Z*^*i*^*)* be the expected cognitive outcome Y for the participant i in the Group *k* conditional on *Z*^*i*^, where *Z*^*i*^ a *p*-dimensional vector of baseline predictors of participant *i*. Here, *μ*_*k*_*(Z*^*i*^*)* was estimated using a random forest model.

A participant’s ITR score, *ITR(Z*^*i*^), was calculated as the difference in the predicted 6-month cognitive score between the treatment and placebo prediction models (the difference of the 6-month cognitive score if a participant were placed under the experimental group versus the placebo group).

$$ITR({Z^i}) = {\mu _{Treatment}}({Z^i}) - {\mu _{Placebo}}({Z^i}),$$

A higher ITR score corresponds to a greater predicted individual treatment benefit, i.e., more improvement on MoCA and CFA associated with the treatment after controlling for the same individual’s placebo effect.

#### Area Between the Curves (ABC) tests of heterogeneity of treatment responses

To determine whether there was heterogeneity in treatment response, we generated an average treatment difference (ATD) curve based on 300 rounds of 3-fold cross-validation, as described below. First, participants in the holdout set were sorted by their ITR scores. Next, we calculated the observed mean difference of 6-month cognitive scores between two randomized groups for the subgroup of participants falling within the upper *q*% ITR score (*q*% ranges from 30% to 98%):

$$E(({Y_{Treatment}} - {Y_{placebo}})\,\,|\,\,ITR(Z) > c),$$

In this equation, *Y* represents the outcome of interest, such as the 6-month MoCA score. *Z* is a matrix of baseline predictors with dimensions *p*^⋆^*n*^*Subgroup*^. *ITR(Z)* is the subgroup ITR score given *Z*^*Subgroup*^, and *c* is a constant value of the average ITR scores for the subgroup that lies within the upper *q*% ITR scores (*c* changes as *q* changes). As the percentage increases, more subjects are included in the percentage subgroup, with 100% representing all participants in the study. The choice of *q* starting at 30% is to ensure a substantial sample size of a minimum of 10 participants for reliable ABC construction (46 ^⋆^ 30% = 14). If there is no response heterogeniety, then the outcome in the above equation remains constant with an overall group mean difference.

By calculating the group mean difference scores involving those that lie within the upper *q%* ITR score, we generated one ATD curve (averaged across 300 rounds of 3-fold cross-validation). With this ATD curve, we could estimate heterogeneity in treatment responses through ABC statistics: the area between the integrals of the observed 6-month group mean difference score (a horizontal curve) and the ATD curve.

#### Permutation tests of heterogeneity of treatment responses

To test the null hypothesis that there is no heterogeneity in treatment responses, we employed permutation tests. Each round of permutation procedure involved the following steps: 1) The original treatment group variable was randomly shuffled to create a new treatment group variable for the entire sample. 2a) For a given participant, if the value of original group variable is the same as the value of new group variable, their new 6-month cognitive score remains the same. 2b) For a given participant, if the value of original group variable is different from the value of new group variable, their new 6-month cognitive score is adjusted by the average treatment effect of the entire sample. This permutation procedure can be described as follows. Let *Y*^*i*^ and *Ŷ*^*i*^ be the original and new 6-month cognitive score of an individual *i, K*^*i*^ and $\widehat {{K^\iota }}$ the original and new group variables of an individual i, and ${\hat \theta }$ be the average treatment effect of the entire sample.

$$\matrix{{\widehat {{Y^\iota }} = {Y^i},\,\,if\,\widehat {{K^\iota }} = {K^i}} \cr {\widehat {{Y^\iota }} = {Y^i} + \hat \theta ,\,\,if\,\widehat {{K^\iota }} = 1\,\& \,{K^i} = 0} \cr {\widehat {{Y^\iota }} = {Y^i} - \hat \theta ,\,\,if\,\widehat {{K^\iota }} = 0\,\& \,{K^i} = 1} \cr } $$

3) One ABC and one ATD were generated through 300 iterations of 3-fold cross-validated random forest models. 4) Step 1 to 3 were repeated for 500 times to generate a range of ABCs and ATDs. 5) We examined the proportion of permuted ABCs that equaled or exceeded the observed ABC under the null hypothesis that observed ABC = 0.

#### Responder characteristics

To identify the variables that most affect treatment responses, we conducted the SHapley Additive exPlanations (SHAP) analysis after 300 iterations of 3-fold cross-validated random forest models (21). The absolute SHAP values provide a measure of the extent to which cognitive outcomes change when a single predictor is added or removed. Next, the top 10 predictors with the highest absolute SHAP values (excluding baseline cognitive score) were included in a full-sample, 2-layer decision tree model to understand the interactions among important predictors, with the outcome being ITR scores.

#### Time saved in cognitive decline

To estimate treatment effects in terms of the potential time saved in cognitive decline, we employed analyses that follow the concept of progression models for repeated measures (PMRM) (16). First, we calculated the group-by-time slopes for MoCA and CFA in the upper 30% ITR participants from baseline to 6 months. Next, we determined the slope of natural cognitive change of the control group. This allowed us to project the expected cognitive slope of the intervention group after 6-month follow-up. We then estimated the time it would take for participants in the intervention group to attain the same cognitive level as those in the control group at 6 months, considering different scenarios where natural cognitive decline of the intervention group is 1 to 5 times faster than the control group. This enabled us to estimate the amount of time that could be saved in slowing down cognitive decline if a participant responds to the treatment.

## Results

A total of 139 participants’ data were analyzed, with 64 in the experimental group and 75 in the control group. The randomization factors included age, sex, years of education, cognitive status (normal vs. MCI) and MoCA score. The baseline characteristics between the two randomized groups showed no significant differences, except for the living arrangement. The control group had a higher number of participants living alone compared to the experimental group (p=0.01) (Table 1).

**Table 1:** Baseline participant characteristics (n=139)

**Characteristics**	**Total**		**Experiment**		**Control**		**t-statistics/*χ***^2^	**p-value**
	**n = 139 (100%)**		**n = 64 (46%)**		**n = 75 (54%)**			
Age [mean (SD)]	80.8	(4.4)	80.5	(4.5)	81.1	(4.3)	t_(137)_=0.82	0.41
Sex (female) [n (%)]	103	(74.1)	47	(73.4)	56	(74.7)	*χ*^2^ = 0.03	0.87
Race (White) [n (%)]	117	(84.2)	55	(85.9)	62	(82.7)	*χ*^2^ = 0.28	0.60
Years of education [mean (SD)]	15.3	(2.2)	15.4	(2.5)	15.1	(2.0)	t_(137)_=−0.72	0.47
Depressive symptoms [mean (SD)]	2.4	(1.8)	2.1	(1.8)	2.6	(1.8)	t_(137)_=1.61	0.11
Mild cognitive impairment [n (%)]	73	(52.5)	33	(51.6)	40	(53.3)	*χ*^2^ = 0.04	0.83
Living alone [n (%)]	90	(64.8)	34	(53.1)	56	(74.7)	*χ*^2^ = 7.02	0.01
Cognitive tests [mean (SD)]								
MoCA	24.1	(3.5)	24.0	(3.5)	24.1	(3.5)	t_(137)_=0.14	0.89
In-person MoCA^†^	23.9	(3.7)	23.7	(3.6)	24.0	(3.8)	t_(103)_=0.33	0.74
Virtual visit MoCA^††^	18.3	(2.5)	18.7	(2.3)	17.9	(2.6)	t_(32)_=−0.95	0.35
CFA	18.6	(4.5)	19.0	(4.6)	18.2	(4.4)	t_(137)_=−1.04	0.30
In-person CFA^†^	18.2	(4.4)	18.5	(4.4)	18.0	(4.4)	t_(103)_=−0.57	0.57
Virtual visit CFA^††^	19.7	(4.8)	20.6	(4.9)	18.9	(4.6)	t_(32)_=−1.02	0.31
Personality [mean (SD)]								
Agreeableness	36.0	(4.9)	36.2	(4.9)	35.9	(4.8)	t_(137)_=−0.42	0.67
Conscientiousness	33.0	(7.2)	33.8	(6.9)	32.4	(7.5)	t_(137)_=−1.08	0.28
Extraversion	25.3	(6.4)	25.3	(6.3)	25.3	(6.5)	t_(137)_=0.07	0.95
Neuroticism	16.4	(8.5)	16.1	(8.7)	16.7	(8.3)	t_(137)_=0.45	0.65
Openness	30.9	(6.6)	31.6	(5.7)	30.3	(7.2)	t_(137)_=−1.23	0.22
Self-reported social time, hours [mean (SD)]								
In-person with family	2.2	(1.7)	2.4	(1.7)	2.1	(1.7)	t_(137)_=−1.08	0.28
In-person with friends	1.8	(1.6)	1.6	(1.6)	1.9	(1.7)	t_(137)_=0.84	0.40
In-person with others	1.2	(1.3)	1.0	(1.2)	1.3	(1.4)	t_(137)_=1.41	0.16
Video chat with family	1.0	(1.1)	1.1	(1.1)	0.9	(1.0)	t_(137)_=−0.81	0.42
Video chat with friends	0.8	(1.0)	0.7	(0.9)	0.8	(1.1)	t_(137)_=0.75	0.45
Text/email with family	0.4	(0.6)	0.3	(0.4)	0.4	(0.6)	t_(137)_=0.80	0.43
Text/email with friends	0.4	(0.6)	0.3	(0.7)	0.4	(0.6)	t_(137)_=0.36	0.72
Time out-of-home	3.9	(1.3)	3.9	(1.3)	3.8	(1.3)	t_(137)_=−0.36	0.72
Self-report general health [mean (SD)]	2.6	(0.8)	2.6	(0.8)	2.7	(0.8)	t_(137)_=0.48	0.63

Note: 139 out of 186 participants had baseline and 6-month cognition data and were included in the analysis. ^†^n=105; ^††^n=34

### Treatment heterogeneity and effects

**Figure 1:**
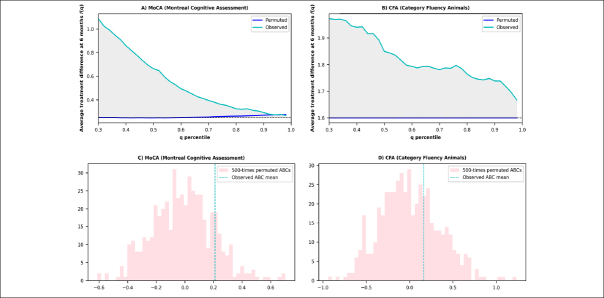
ATD and ABC plots from observed data versus permuted data Note: A) The ATD curves depict the comparison between observed and permuted data for the MoCA. The grey area corresponds to the observed ABC value of 0.21. In the absence of heterogeneity, the ATD curve would mirror the permuted line, depicting consistent treatment differences between the placebo and experimental groups across all q percentiles (i.e., ABC=0). B) The ATD curves depict the comparison between observed and permuted data for the CFA. The grey area represents an observed ABC value of 0.16. In the absence of heterogeneity, the ATD curve would mirror the permuted line, depicting consistent treatment differences between the placebo and experimental groups across all q percentiles (i.e., ABC=0). C) The histogram displays 500 repeats of permuted ABCs for the MoCA. Each ABC value was generated through 300 rounds of 3-fold cross-validation. The proportion of ABCs equal to or exceeding the dashed line indicates the probability of the observed ABC rejecting the null hypothesis of ABC = 0. D) The histogram displays 500 repeats of permuted ABCs for the CFA. Each ABC value was generated through 300 rounds of 3-fold cross-validation. The proportion of ABCs equal to or exceeding the dashed line indicates the probability of the observed ABC rejecting the null hypothesis of ABC = 0.

For MoCA, the average of ABCs of observed dataset and permuted dataset were 0.21±0.68 and 0.0±0.21, respectively (Figure 1). MoCA results suggested substantial treatment response heterogeneity, with only 15% of permuted results falling beyond the observed ABC value of 0.21 under the null hypothesis of observed ABC=0. For CFA, the average of ABCs of observed dataset and permuted dataset were 0.16±0.97 and 0.0±0.34, respectively (Figure 1). CFA results suggested a modest probability of response heterogeneity, with 32% of permuted results falling beyond the observed ABC value of 0.16 under the null hypothesis of observed ABC=0.

Participants with the upper 30% ITR scores would exhibit a 1.2 points difference on the 6-month MoCA score between the two randomized groups. In contrast, for all participants, irrespective of their ITR score, the 6-month MoCA score difference between the two randomized groups (treatment - control) was estimated as 0.25 points (MCI group: 1.26; Cognitive normal group: −0.98) (Figure 1).

For CFA, participants with the upper 30% ITR scores would have a 1.92 points difference between the two randomized groups, while for all participants, the 6-month CFA score difference between the two randomized groups (treatment - control) would be 1.58 points (MCI group: 0.93; Cognitive normal group: 2.18) (Figure 1).

### Treatment responder profiles

**Figure 2:**
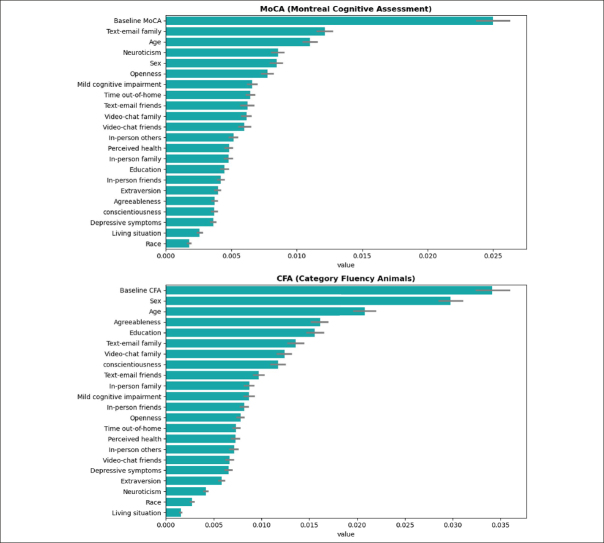
SHAP analysis and values

From the SHAP analysis of MoCA, age, cognitive status (MCI vs. normal), social time with family, sex, and neuroticism personality were identified as important features determining treatment responses (Figure 2). For CFA, age, cognition, sex, and social time with family, and social time with friends were identified as important features determining treatment responses (Figure 2).

**Figure 3:**
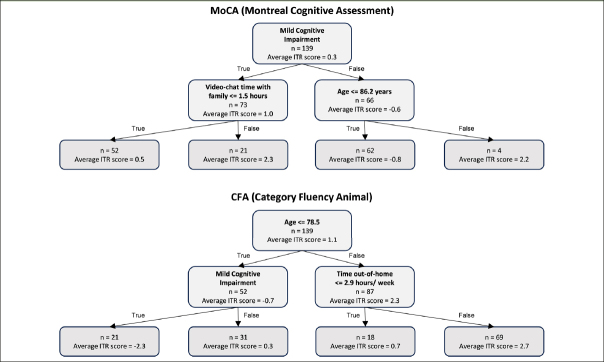
Decision tree models with the outcome being ITR scores

The full-sample decision tree model of MoCA revealed that 18% of the participants (n = 25), who were both MCI (vs. normal cognition) and spent at least 1.5 hours per week of in-person time with family, gained the most benefit from the intervention, as indicated by the highest average ITR score. On the other hand, around 45% of the participants (n = 62) who were cognitively normal and younger were less likely to benefit from the intervention, as indicated by a negative average ITR score (Figure 3).

The full-sample decision tree model of CFA revealed that 50% of the participants (n = 69), who were aged > 78.5 years old and spent at least 2.9 hours out-of-home per week, gained the most benefit from the intervention, as indicated by the highest average ITR score. On the other hand, around 15% of the participants (n = 21) who were cognitively impaired and younger were less likely to benefit from the intervention, as indicated by a negative average ITR score (Figure 3).

### Time saved in cognitive decline among top 30% of responders

**Figure 4:**
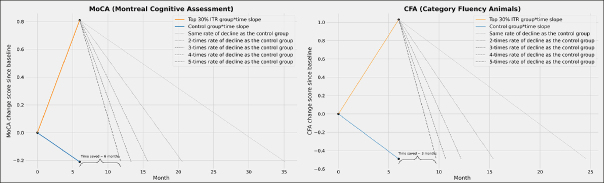
Proof of concept of estimated time saved in cognitive decline Note: The slopes of the control group were estimated using those who completed the in-person MoCA at both baseline and 6-month follow-up.

We estimated the time it would take for participants in the intervention group to attain the same cognitive level as those in the control group at 6 months, considering different scenarios where natural cognitive decline of the intervention group is 1 to 5 times faster than the control group. For MoCA, those who were allocated to the intervention group and were in the upper 30% ITR could benefit from a time saved in cognitive decline for 6 months if their natural cognitive decline after intervention is 5-times faster than the control group (Figure 4). For CFA, those who were allocated to the intervention group and were in the upper 30% ITR may benefit from time saved in cognitive decline of up to 3 months if their natural cognitive decline after intervention is 5-times faster than the control group (Figure 4).

## Discussion

In this paper, we describe an individual-level treatment response approach to quantify the heterogeneity of treatment responses and gauge the potential time saved in cognitive decline arising from a conversational engagement intervention. In contrast to the conventional responder analysis that solely considers a person’s randomized group assignment, the current approach estimates the potential gain or loss of the outcome if a person could be placed in both experimental and control groups. This approach evaluates the standardized individual mean differences within randomized groups, offering a reliable way of estimating heterogeneity of treatment responses in clinical trials. The time saved with treatment provides a proof of concept into the clinical meaningfulness of I-CONECT. This series of analyses provides a rigorous method of exploring the efficacy and clinical relevancy of AD clinical trials.

### Treatment heterogeneity and responder characteristics

Treatment heterogeneity testing is the first step in determining whether participants respond differently to the treatment (22). In the I-CONECT trial, the heterogeneity of treatment responses was observed using the ABC statistics and permutation tests, implying that the effect of conversational engagement varies across participants in terms of MoCA. The treatment heterogeneity was relatively modest in CFA. We further identified participant characteristics that could influence treatment effects. By calculating the contribution of each feature to the prediction using SHAP analysis, we found age, social time, and cognitive status are the key determinants of I-CONECT effectiveness. Responders of I-CONECT tend to have MCI and spend some level of time with family when the intervention outcome is MoCA (global cognition). With CFA (semantic fluency) as the intevention outcome, responders tend to be on the better health spectrum (i.e., younger old individuals who are cognitively normal and spend more time out-of-home). This suggests that a behavioral intervention like I-CONECT might be suitable for multiple treatment purposes such as supporting healthy aging and delaying further cognitive decline among those with MCI. The result further confirms our previously reported topline results (13) where we showed gains in MoCA among the MCI and gains in CFA among those with normal cognition through the intervention. The full-sample decision tree offers an approach to investigating the interplay of important participant characteristics. This contemporary machine learning technique is well-suited for responder profiling for future trial design and enrichment.

### Clinical meaningfulness – time saved in cognitive decline

Besides identifying promising subgroups, we also quantified the clinical meaningfulness of the I-CONECT intervention. Conventional mixed model for repeated measures (MMRM) obtain the score differences between randomized groups at various time points of follow-ups. This approach is often criticized as difficult to interpret in terms of clinical meaningfulness. The PMRM concept proposes estimating the time saved with treatment between randomized groups. Although our follow-up time points (baseline; 6 months) were not enough for a PMRM analysis, we adopted the concept and projected the anticipated slopes of cognitive changes after 6-month follow-up based on the dementia disease modification framework (23). We took a conservative approach, assuming the rate of cognitive decline in the treatment group after 6-months would be 1–5 times faster than the control group (natural decline from baseline to 6-month). Intervention group participants in the upper 30% of ITR scores demonstrated potential delays of 3 months in semantic fluency (CFA) and 6 months in global cognition (MoCA), assuming a 5-fold faster natural cognitive decline compared to the control group post-treatment. The time saved in cognitive decline brought by social stimulation has clinical meaningfulness and economic benefits, especially considering the cost associated with dementia care. For example, leveraging national representative surveys, projections suggest that a 5-year delay in the AD onset could result in 23% reduction in informal care cost (24). While such projections must be interpreted with caution based on our small dataset of course, this proof-of-concept analysis provides a theoretical framework and method for evaluating the potential benefit of an intervention for particular persons.

### Study limitations

We acknowledge several limitations in our study design. Our sample size is limited. To alleviate over-fitting, we splitted the dataset into training (n=93) and testing/holdout datasets (n=46) for the random forest model. The ABC statistics were constructed using the small sample size holdout set (e.g., 30%, n=46). Therefore, we ran 300 rounds of the 3-hold cross validation random forest models to quantify the uncertainty of the ABC statistics. Still, the small sample size for model development and ABC statistics may affect the reliability of our findings. In our sequence of analyses, the same data used to derive the top 30% of the subpopulation were used to assess the time saved for cognitive decline. This could potentially lead to selection bias and result in over-estimation of treatment effects. Furthermore, our analyses encompassed both MCI and cognitively normal participants, which could potentially dilute the treatment effects observed in our topline results paper. Of note, we focused only on cognitive outcomes and did not investigate functional outcomes such as instrumental activities of daily living (IADL) scores, so extrapolating the results to prevent AD progression may not be appropriate. In terms of prediction models, we primarily relied on random forest for constructing the ITR scores, though alternative models like the least absolute shrinkage and selection operator (LASSO) may also be effective in constructing ITR scores. Additionally, our analysis could have benefited from the inclusion of other data sources, such as neuroimaging, cerebrospinal fluid markers, and genetic information, to provide potential biological explanations for responders (25). Another challenge in the current study was to delineate natural cognitive slope or trajectory (26). Therefore, we took a conservative approach to projecting anticipated slopes after intervention to estimate the time saved for cognitive decline in responders.

### Future directions

Any given pharmacological or behavioral intervention might be suitable for some but not all. Sophisticated methods for profiling treatment responders can lead to improved therapy, guiding selection of treatment for patients most likely to respond. We hope to leverage insights derived from the ITR, SHAP, and decision tree analyses in framing inclusion and exclusion criteria for the next I-CONECT trial. In forthcoming clinical trials, we want to expand the range of follow-up time points to understand the long-term economical and health benefits of I-CONECT.

## Notes

*Ethical standards:* The I-CONECT study was approved by the institutional review board (IRB) at the Oregon Health & Science University (OHSU) (STUDY00015937).
